# Empathy in Chinese eight-year medical program students: differences by school year, educational stage, and future career preference

**DOI:** 10.1186/s12909-018-1348-2

**Published:** 2018-10-22

**Authors:** Dongju Li, Huiming Xu, Mingyi Kang, Shulan Ma

**Affiliations:** 1 Experimental Teaching Center of Basic Medicine,School of Basic Medical Sciences, Fudan University, Shanghai, China; 2 Shimen Second Road Community Health Service Center, Jing-An District, Shanghai, China; 3 Department of Integrative Medicine and Neurobiology, School of Basic Medical Sciences, Fudan University, Shanghai, China

**Keywords:** Empathy, Chinese eight-year medical students, Jefferson scale of empathy

## Abstract

**Backgroud:**

To examine the psychometric properties of a Chinese translation of the Jefferson Scale of Empathy (Student-version, JSE-S), and to study differences in empathy scores among eight-year undergraduate medical students across gender, year of study, and future career preference.

**Methods:**

The JSE-S was administered to 442 participants from December 2016 to July 2017, who were all first- to seventh-year students on an eight-year medical education course at Fudan University. Factor analysis was used to examine the underlying components of the Chinese version of the JSE-S. The data analyses comprised a t-test and analyses of variance.

**Results:**

Factor analysis confirmed four components: perspective taking, compassionate care, ability to stand in patient’s shoes, and difficulties in adopting patient’s perspective. The lowest empathy score was found in the seventh-year students (99.5), while a decline was found across school years. Students in clinical training (sixth/seventh year) had lower empathy than students in premedical study (first/second year), basic medicine (third/fourth year), and clinical medicine (fifth year). Statistically significant differences in empathy mean scores were found in respect of future career preference but not gender. Students who preferred not to become doctors had lower empathy than students who preferred to become doctors, who were undecided, and who did not specify.

**Conclusions:**

The findings support the construct validity and reliability of the Chinese version of the JSE-S for medical students. The study also revealed the features of empathy in eight-year program students, and provided a reliable reference to design interventions to cultivate empathy among Chinese medical students.

## Background

While the patient-physician relationship stands at the very heart of medicine, the poor relationship between patient and physician has become prominent in China [1, 2] and has been deteriorating over the past 20 years [3, 4]. Many complex reasons contribute to a tense patient-physician relationship, but the lack of communication skills amongst physicians is an important factor [5]. Empathy is a crucial component in patient-physician interactions and has been associated with improved patient compliance, satisfaction, and clinical outcomes, as well as with decreased provider burn-out and litigation [6–9]. Since the discovery of the beneficial effects of empathy in medical practice, many efforts have been made to define and evaluate it, but the most widely recognized definition of cognitive empathy in the context of patient care is that proposed by Hojat and his colleagues at Jefferson Medical College [10, 11]. The Jefferson Scale of Empathy (JSE) is a reliable instrument that has been specifically designed to assess empathy in health professionals (HP-version) and medical students (S-version). It has been translated into 54 languages, and used in 80 countries worldwide [12]. A number of studies have observed empathy using the JSE-S among medical students from different countries, and empathy scores in terms of gender, school year, and future career specialty have been the emphases of most studies [13–18].

In modern-day China, expectations regarding the country’s healthcare system have risen. To meet these growing societal demands, a major reform of how physicians are trained has resulted in the widespread implementation of an eight-year medical education program throughout China in the past decade [19]. From 2001 to the present, 18 leading medical schools in China have created an eight-year medical education program [19], designed to train health professionals to the highest standards [20]. Students who complete the eight years of medical education receive a doctoral degree in medicine, and generally, medical graduates from the eight-year program work in the nation’s top hospitals, with most ascending to significant leadership roles through career advancement. As future medical leaders, the empathy levels of eight-year program students have become the central focus of educational efforts. To the best of our knowledge, however, no previous study has evaluated the empathy levels of Chinese eight-year medical education program students. In this study, therefore, we aim to examine the factor structure of a Chinese version of the JSE-S with Chinese students, as well as to assess the differences in empathy scores by gender, year of study, and future career preference in medical students of the eight-year program.

## Method

### Participants

This research comprised a cross-sectional study using a paper-based questionnaire, of students enrolled in the eight-year medical education program at Fudan University, China. The eight-year program consists of two years of theory-based training in general sciences and humanities, followed by a six-year medical training, which is divided into two years of basic medicine, one year of clinical medicine, one year of internship, and two years of clinical rotations. In this study, participants ranged from the first to seventh years of the eight-year program; eighth-year students were excluded as they are dispersed to different regions for clinical rotations, which makes it difficult to collect sufficient effective samples. Given the teaching plan, participants were categorized into four educational stages: premedical study (first and second year), basic medicine (third and fourth year), clinical medicine (fifth year), and clinical training (sixth and seventh year). The Chinese student version of the JSE-S was distributed to 550 medical students on the eight-year program at Fudan University between December 2016 and July 2017. This study was approved by the university’s research ethics committee. Students were not compensated for their participation.

### Instrument

The JSE-S version includes 20 items answered on a 7-point Likert scale (1 = strongly disagree, 7 = strongly agree). Ten of the items are positively worded and 10 negatively worded. All the 20 items were listed in the Table 1. The JSE-S total possible score ranges from 20 to 140 with higher values indicating a higher level of empathy. Satisfactory evidence has been tendered in support of the psychometric properties of this scale [13, 17, 21, 22].

**Table 1:** Frequency and percentage distributions and descriptive statistics of scores on the JSE-S by gender

Score interval	Male (*n* = 201)			Female (*n* = 241)			Total(*n* = 442)		
	frequency	cumulative frequency	percentile ranks	frequency	cumulative frequency	percentile ranks	frequency	cumulative frequency	percentile ranks
≤80	12	12	6%	11	11	5%	23	23	5%
81–85	12	24	7–12%	5	16	5–7%	17	40	6–9%
86–90	9	33	13–16%	5	21	8–9%	14	54	10–12%
91–95	15	48	17–24%	19	40	10–17%	34	88	13–20%
96–100	29	77	24–38%	33	73	20–30%	62	150	20–34%
101–105	37	114	43–57%	46	119	32–49%	83	233	37–53%
106–110	33	147	62–73%	44	163	52–67%	78	311	53–70%
111–115	17	164	74–82%	31	194	72–81%	48	359	73–81%
116–120	20	184	83–92%	16	210	81–87%	35	394	82–89%
121–125	10	194	93–97%	20	230	88–95%	30	424	91–96%
126–130	6	200	97–100%	6	236	96–98%	12	436	97–99%
> 130	1	201	100%	5	241	98–100%	6	442	99–100%
Mean score (SD)^a^	102.8 (13.5)			105.3 (13.7)			104.2 (13.6)		
Median score	103.2			105.6			104.8		
Possible range	20–140			20–140			20–140		
Actual range	38–134			34–136			34–136		

a The mean empathy scores were compared between male and female students by t-test and the *p*-value is 0.058, which is not significant at the 0.05 level

### Procedures

We explained the purpose of the study to the participants, and also informed them that participation was voluntary and anonymous. Students took approximately 15 min to complete the questionnaire, and implied their consent by its completion and submission.

### Statistical analyses

We used Pearson correlation coefficients to examine item-total score correlations. We used factor analysis (principal component factor extraction) with varimax rotation to search for the underlying factor structure of the Chinese version of the JSE-S. We then calculated the Cronbach’s alpha coefficient to assess the internal consistency aspect of the reliability of the questionnaire. We compared empathy scores between male and female students using a t-test, and used analysis of variance (ANOVA), including post hoc tests, to compare empathy scores across school years, categorized stages, and future career preference. We performed statistical analysis in SPSS (IBM, Armonk, NY, USA), with the significance level set at *p* < .05.

## Results

### Response rate

We distributed a total of 550 questionnaires to medical students on the eight-year education program in Fudan University, China, of which 458 questionnaires were returned, for a response rate of 83%. Of these, we excluded 16 questionnaires from the sample due to incomplete demographic data. Consequently, we included 442 questionnaires in the final analysis. The sample comprised 201 male students (45%), and 241 female students (55%). The Cronbach’s alpha coefficient in our study was 0.87.

### Score distributions and percentile ranks

The frequency distributions of the JSE-S scores and percentile ranks for men, women, and the entire sample are presented in Table 1. Gender differences were analyzed using independent samples *t*-tests, but no gender differences on the JSE-S mean scores appeared.

### Underlying factors

As shown in Table 2, four factors emerged, each with an eigenvalue greater than one, accounting for a total of 53% of variance before rotation. Factor 1, which accounted for 24% of the variance, is a major component that can be labeled “perspective taking” based on the content of the 10 items with factor coefficients greater than 0.5. Factor 2 can be considered “compassionate care” based on the content of six items with factor coefficients greater than 0.5. Factors 3 and 4 were trivial, with factor coefficients greater than 0.5 on only two items. Factor 3 may be considered “ability to stand in patient’s shoes,” while Factor 4 is similar to the factor “difficulties in adopting patient’s perspective.”

**Table 2:** Rotated factor coefficients by item means, standard deviations, and item–total score correlations of the JSE-S

Item†	Factor 1	Factor 2	Factor 3	Factor 4	Mean (SD)	r_it_
Physicians should try to think like their patients in order to render better care. (17)	**.72**	.15	−.09	.05	5.3 (1.3)	.61^**^
Physicians should try to stand in their patients’ shoes when providing care to them. (9)	**.71**	.08	.21	−.01	5.5 (1.3)	.63^**^
Physicians’ understanding of the emotional status of their patients, as well as that of their families, is one important component of the physician–patient relationship. (16)	**.70**	.24	−.02	.28	5.7 (1.1)	.67^**^
I believe that empathy is an important therapeutic factor in the medical treatment. (20)	**.66**	.24	.03	.25	5.5 (1.2)	.66^**^
Understanding body language is as important as verbal communication in physician–patient relationships. (4)	**.61**	.09	.17	.09	5.8 (1.2)	.57^**^
Patients feel better when their physicians understand their feelings. (2)	**.61**	.06	.04	.14	6.0 (1.1)	.53^**^
A physician’s sense of humor contributes to a better clinical outcome. (5)	**.60**	.04	.23	.12	5.4 (1.4)	.57^**^
Physicians should try to understand what is going on in their patients’ minds by paying attention to their nonverbal cues and body language. (13)	**.58**	.21	.05	−.08	5.3 (1.2)	.55^**^
Patients value a physician’s understanding of their feelings which is therapeutic in its own right. (10)	**.56**	.22	.09	−.06	5.5 (1.2)	.56^**^
Empathy is a therapeutic skill without which the physician’s success is limited. (15)	**.55**	.19	−.21	.09	4.6 (1.5)	.50^**^
Physicians’ understanding of their patients’ feelings and the feelings of their patients’ families does not influence medical or surgical treatment. (1)	−.03	**.68**	.01	−.02	5.2 (1.6)	.38^**^
Asking patients about what is happening in their personal lives is not helpful in understanding their physical complaints. (12)	.28	**.64**	.13	.30	5.5 (1.2)	.62^**^
Attentiveness to patients’ personal experiences does not influence treatment outcomes. (8)	.19	**.61**	.03	−.24	5.0 (1.5)	.46^**^
Patients’ illnesses can be cured only by medical or surgical treatment; therefore, physicians’ emotional ties with their patients do not have a significant influence in medical or surgical treatment. (11)	.46	**.60**	.11	.22	5.6 (1.2)	.71^**^
I believe that emotion has no place in the treatment of medical illness. (14)	.48	**.55**	.13	.15	5.6 (1.3)	.70^**^
Attention to patients’ emotions is not important in history taking. (7)	.42	**.52**	.21	.17	5.8 (1.2)	.67^**^
It is difficult for a physician to view things from patients’ perspectives. (3)	.10	.05	**.86**	.06	4.1 (1.5)	.39^**^
Because people are different, it is difficult to see things from patients’ perspectives. (6)	.13	.20	**.85**	−.07	4.3 (1.5)	.48^**^
I do not enjoy reading nonmedical literature or the arts. (19)	.06	.20	.17	**.71**	5.8 (1.4)	.36^**^
Physicians should not allow themselves to be influenced by strong personal bonds between their patients and their family members. (18)	−.20	.12	.20	**−.64**	2.7 (1.4)	−.05
Eigenvalue	4.78	2.53	1.82	1.38		
% Variance	24	13	9	7		

† Items are listed by the order of magnitude of the factor coefficients within each factor. Factor loadings equal to or greater than 0.4 are in bold. Numbers in parentheses represent the sequence of the items in the actual scale. Items were scored using a seven-point Likert-type scale

*r*_it_ = Item-total score correlation. ** Indicates statistical significance levels *p* < .01

### School year difference in JSE-S scores

We performed an ANOVA to explore the effect of school year on empathy mean scores. As shown in Table 3, the mean empathy scores declined from 107.6 in the first year to 104.1 in the fourth year. An elevated score appeared in the fifth year (109.1) and the decline continued to 99.5 in the seventh year. We detected a statistically significant difference in the JSE-S mean scores in different school years (*p* < .001). There was a difference between fifth- and sixth-year students (109.1 vs 101.2, *p* = .035), which corresponded to the first clinical training year in medical school. The seventh-year students had significant differences in empathy scores when compared to the first-year students (99.5 vs 107.6, *p* = .007), and seventh-year students differed from fifth-year students (99.5 vs 109.1, *p* = .002).

**Table 3:** Changes in mean scores on the JSE-S throughout the medical school years

Medical school year	Male		Female		Total	
	Number	Mean score (SD)	Number	Mean score (SD)	Number	Mean score (SD)
First-year	32	106.5 (9.7)	30	108.8 (17.4)	62	107.6 (13.9)^**^
Second-year	17	108.2 (11.2)	32	105.0 (15.2)	49	106. (13.9)
Third-year	35	102.7 (10.1)	35	107.2 (11.5)	70	105.0 (11.0)
Fourth-year	27	102.3 (10.6)	34	104.7 (10.3)	61	104.1 (14.4)
Fifth-year	20	104.3 (15.0)	26	112.7 (9.3)^†^	46	109.1 (12.7)^**^
Sixth-year	39	100.4 (14.5)	36	102.0 (10.2)	75	101.2 (12.6)^#^
Seventh-year	32	99.5 (14.4)	47	99.4 (15.1)	79	99.5 (14.7)^##^

**P < .001 compared to the Seventh-year, # *P* < .05, ## *P* < .01 compared to the Fifth-year

† *P* < .05 compared to the male in the same school year

We also compared empathy scores between cohorts in different years for each gender using a t-test. Except for 5th year, there was no significant gender difference in respect of the other school years. There was a significant gender difference in respect of year 5th (104.3 vs 112.7, *p* = 0.035).

### The effect of educational stage difference on JSE-S scores

**Fig. 1:**
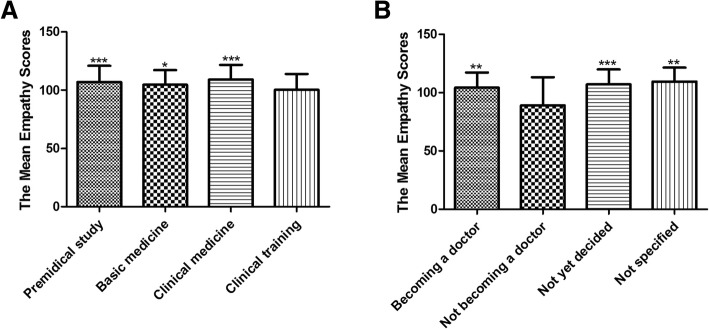
The differences in empathy mean scores in terms of categorized stages and future career preference. **a**: Empathy trends in educational stages were examined using ANOVA. Premedical study comprises two years of general education during school years 1 and 2 (*n* = 111). Basic medicine comprises two years of basic medicine study during school years 3 and 4 (*n* = 131). Clinical medicine comprises one year of clinical medicine study in school year 5 (*n* = 46). Clinical training comprises two years of internship and clinical rotations during school years 6 and 7 (*n* = 154). We found statistically significant associations between changes in empathy scores and educational stages (*p* = .001). There was a difference in JSE-S scores between students in clinical training and premedical study, between those in clinical training and basic medicine, and between those in clinical training and clinical medicine.* *p* < .05, *** *p* < .001 compared to the clinical training. **b**: We analyzed the empathy mean scores in future career preference using ANOVA. There are three options for future career preference: becoming a doctor (*n* = 380), not becoming a doctor (*n* = 11), and not yet decided (*n* = 37). Participants who did not specify their preference were included as a fourth option: not specified (*n* = 14). Statistically significant differences in the empathy mean scores were found by future career preference (*p* = .001). Students who preferred not to become doctors had lower empathy than those who did prefer to become doctors, those who were undecided, and those did not specify.** *p* < .01, *** *p* < .001 compared to not becoming a doctor

Participants were categorized into four educational stages: premedical study (years 1 and 2), basic medicine (years 3 and 4), clinical medicine (year 5), and clinical training (years 6 and 7). We examined the empathy trends in educational stages using ANOVA and found a statistically significant association between changes in empathy scores and educational stages (*p* = .001, Fig.1a). Students at the clinical medicine stage had the highest empathy scores (109.1), while those in clinical training had the lowest empathy scores (100.3). There was a difference in JSE-S scores between students in clinical training and premedical study (100.3 vs 107.0, *p* = .0004), between those in clinical training and basic medicine (100.3 vs 104.5, *p* = .042), and between those in clinical training and clinical medicine (100.2 vs 109.2, *p* = .001).

### The effect of future career preference on JSE-S scores

Due to the tense patient-physician relationships in Chinese hospitals, the survey also included a set of questions to compare mean differences in empathy scores based on future career preference (options: becoming a doctor, not becoming a doctor, not yet decided). In total, 428 (97%) participants declared their future career plans. We also included the 14 (3%) participants who did not specify their plans as a fourth option (not specified). We found a statistically significant difference in empathy mean scores according to future career preference (*p* = .001, Fig.1b). Students who preferred not to become doctors had lower empathy than those who did prefer to become doctors (89.1 vs 104.1, *p* = .002), those who were undecided (89.1 vs 106.9, p = .001), and those did not specify (89.1 vs 109.4, p = .001).

## Discussion

Following its release of the two policies “Opinions on Medical Education Reform” and “Opinions on implementing excellent physician education program” in 2012, the Chinese Ministry of Education standardized medical education training programs to three types – three-year, five-year, and eight-year programs, all of which have different training goals. The eight-year curriculum is designed to train health professionals to the standard of an MD or PhD degree and is only offered in leading medical schools in China [19]. Chinese medical students are recruited directly from high school after passing a National College Entrance Examination. Usually, the applicants to the eight-year program must have a much higher score than those to the three-year and five-year programs. The students enrolled in the eight-year program at Fudan University (one of the top medical schools in China) usually rank in the top 1% of their high school graduating classes. In this study, we used the Chinese version of the JSE-S to reveal the level of empathy among Chinese medical students on the eight-year education program and reported the results of factor analysis for the 20 items of the JSE-S amongst the eight-year program students. The grand factor of “perspective taking” emerged and we described this component as a major dimension of empathy in patient care [23]. Two other components of empathy also emerged, i.e., compassionate care and standing in the patient’s shoes, which are both specific to the patient-physician relationship. These three factors also emerged in American [23, 24], Japanese [21], and Mexican medical students [25], as well as Chinese medical students on the five-year program [22]. Another factor, “difficulties in adopting patient’s perspective,” also appeared in the eight-year program students. While this factor was also found in Japanese samples [21], it was not found in five-year program Chinese samples [22]. This difference between Chinese medical students might be attributed to the student pool in various Chinese universities and the varying program curriculums.

In this study, we also found that changes in empathy were unrelated to gender, despite female students scoring slightly higher than males (105.3 vs. 102.8, *p* = .058; Table 1). Although our results differed from studies on medical students published in America [14, 15], Mexico [25], the UK [16, 26], Australia [27], and Japan [21], they were consistent with published studies in Iran [28], America [13, 29], and Korea [30–32]. The conflicting results are considered to stem from cultural and social differences. For example, a factor in Chinese schools is that female teachers outnumber their male counterparts, predominating in the classroom from kindergarten to higher education. This could cause students to miss out on male role models and reduce their opportunities to address the gender gap [33]. An interesting result of our study was that the gender difference in empathy was inconsistent between eight-year program medical students and their five-year program counterparts. Sociocultural factors, such as gender stereotypes and gender socialization, may contribute to the difference in empathic tendencies between girls and boys [27, 34]. Culturally stereotyped sex differences in desired or expected behavior may lead parents to foster divergent developmental outcomes in their daughters and sons [35], in promoting the nurturing, caring, and sensitive side of girls and the more assertive, independent, and active side of boys. Children perceive these gender stereotypes and expectations about them and take up corresponding roles and responsibilities, which develop and reinforce their behavior as they grow up. The one-child policy in China, however, has changed the composition of the family, an important part of Chinese culture. These changes in the family pattern have challenged the traditional attitudes and expectations associated with the respective gender roles. Girls enrolled in the eight-year program have gone through a highly competitive process from elementary school to high school and finally outperform others to emerge on top in the university entrance examination. These experiences foster the more assertive, independent, and active side of girls. In light of this, gender stereotypes and expectations become complicated for straight A students in modern China. Gender gaps in empathy that are attributed to socio-cultural factors are becoming fuzzy among the eight-year program students. This might explain why gender differences are inconsistent among Chinese medical students between the five-year and eight-year programs. This inconsistency among Chinese medical students indicates that more studies are required to explore whether or not women are more empathetic than men.

The eight-year medical curriculum in China differs from those in the United States and Japan, as well as the five-year program in China. The eight-year program at Fudan University consists of a two-year theory-based training in general sciences and humanities, followed by a six-year medical training. In the present study, there was a downward trend in empathy among medical students as they progressed through medical school. This decline began from the sixth year, which corresponds to the first clinical training year and continued to the seventh year. As the sixth and seventh years comprise the medical training years, we further categorized the students into four educational stages to better understand the decline in empathy during medical school training. We found a significant decline in JSE-S scores in clinical training students, which further confirmed that empathy is known to decline, rather than grow, following clinical training. A number of studies have observed a reduction in empathy during the final training courses. For example, a statistically significant decline in empathy amongst American medical students following the first full year of clinical experience has been reported in both cross-sectional and longitudinal studies [13–15]. Generally, student doctors of the 8-year program engage in clinical training in national hospitals, in which patient overload, low doctor-to-patient ratios, and short patient consultations result in a decreased quality of patient care. The application of highly sophisticated medical equipment in diagnosis and treatment aggravates this situation. The more doctors depend solely on technology, the more they lose their humanism. During clinical training, eight-year program student doctors must deal with various stressors, including personal safety, intense patient-physician relationships, as well as work-related challenges, including long work hours and sleep deprivation, all reasons believed to contribute to the decline in their empathy [1, 2, 36, 37]. Another possible explanation for the observed decrease in empathy might be a sense of elitism. Students’ identification with a cold and uncaring role model, greater emphasis on technological than humanistic aspects of medicine, and the developing sense of being part of an elite group are amongst the factors that contribute to a decline in empathy during medical education [38]. Eight-year program students usually rank in the top 1% of high school graduates, and enjoy the highest standard of medical training, which may easily foster a sense of elitism and lead to deterioration of empathy. On the other hand, this downward trend also suggests that empathy could be amenable to a range of educational interventions during medical school [34, 35], which might explain why empathy scores increased in other studies as students progressed through medical school [21, 27, 33]. Further research is required to identify factors that contribute to changes in empathy and to examine whether targeted educational strategies could help to retain, reinforce, and cultivate empathy among medical students.

In China, the eight-year medical education program is a medical elite talent training model, and graduates thereof are favored by hospitals, research institutions, the pharmaceutical/biotech industries, government, and private organizations. Considering the tense patient-physician relationships in Chinese hospitals, we were interested in the future career preference of medical students. The mean empathy score of students who preferred not to become doctors was the lowest, and much lower than the mean score of other options. Because those who begin medical school with the lowest empathy have a steeper decline in empathy than those initially in the highest tier [14], in this cross-sectional study we were unable to confirm whether low empathy scores were due to an initial low score or the result of decline. Thus, we further analyzed the school year distributions of the students who chose not to become doctors. The empathy scores of students in the two premedical years were higher than those of students in the six medical years (107.5 vs 78.6, *p* = .048, data not shown). Thus, the lower empathy scores of students who preferred not to become doctors might be attributable to a decline during the medical school years. The two-year premedical curriculum is in general sciences and humanities. During the following six years, the prioritization of acquiring medical expertise over humanistic knowledge leads students to concentrate heavily on medical knowledge and ignore the humanistic value of medicine. Furthermore, the teaching of empathy is poorly covered in medical teaching plans, which might aggravate the decline in empathy. Moreover, previously, a survey on future career plans was conducted on medical students in the five- and eight-year programs at Fudan University, which showed that some medical students even preferred careers totally unrelated to medicine [39]. Thus, there is a conflict as to whose career goal students are actually fulfilling, which might lead to a decline in empathy as an acculturation phenomenon [15].

We must also acknowledge that the participants were among the top level of medical students in China, we cannot claim that our sample was representative of empathy levels among medical undergraduates countrywide. We also employed a self-reporting scale of empathy and, although these scales have been reported to be reliable and valid, there are possible discrepancies between self-report and actual behavior and the self-reports might have been subjected to bias. Furthermore, our findings were based on a cross-sectional design, and the possibility of cohort effects could not be dismissed in our study. Thus, a longitudinal study is recommended to verify the findings. Finally, since the medical students came from across the country, their family backgrounds might differ greatly, such as living in a joint family or nuclear family, parents’ education levels and household income [40, 41], which may have influenced their empathy scores.

## Conclusion

In this study, we focused on the empathy levels of eight-year program medical students who are considered the beneficiaries of the highest standard of medical training and the future backbone staff in hospitals in China. We confirmed three factors of “compassionate care,” “perspective taking,” and “ability to stand in patient’s shoes,” as well as significant changes in empathy over time. We also demonstrated that empathy typically decreases following the full clinical training years and found statistically significant differences in the mean empathy scores based on future career preference among the eight-year program students. Our findings call on medical educators to reconsider what interventions can be designed to mitigate the negative impact of clinical training on empathy and whether the highest standard educational program can help nurture empathy among medical students.

## Abbreviation

JSE-S: The Student Version of the Jefferson Scale of Empathy
